# Disruption in Proteostasis, Microglia‐mediated Synapse pruning, and Neuronal populations in BSN P3866A knock‐in Mouse model of Neurodegeneration

**DOI:** 10.1002/alz70855_105833

**Published:** 2025-12-24

**Authors:** Henika Patel, Pablo Martinez, Daniella Lopes, Enrique Chimal Juarez, Nur Jury, Katie Vanderbosch, Yanwen You, Israel Coronel, Luke C. Dabin, Miguel Moutinho, Jungsu Kim, Melissa E. Murray, Cristian A Lasagna Reeves

**Affiliations:** ^1^ Baylor College of Medicine, Houston, TX, USA; ^2^ Indiana University School of Medicine, Indianapolis, IN, USA; ^3^ Mayo Clinic, Jacksonville, FL, USA

## Abstract

**Background:**

Neurodegenerative tauopathies, characterized by the aggregation of misfolded tau protein, poses a significant clinical and scientific challenge. Tau interaction with other proteins affects the formation of ‘tau‐seed’ but less is known about its effects on tau‐seed properties. Our previous study revealed that Bassoon (BSN), a presynaptic protein, interacts with tau‐seed, exacerbating its toxicity. A parallel study also associated missense mutations in *BSN* with tau aggregation in patients, prompting an investigation into the influence of BSN and its mutations in neurodegeneration for potential therapeutic insights.

**Method:**

We generated a knock‐in mouse model (BSNKI) harboring the disease‐associated p.Pro3866Ala mutation in endogenous Bsn. Cognitive and motor abilities were evaluated throughout the lifespan of heterozygous and homozygous BSNKI mice, along with analyses of BSN and tau patterns, gliosis, and gene expression changes in their brains. Additionally, we conducted single‐nucleic RNA sequencing to identify changes at the cellular level.

**Result:**

BSNKI mice displayed progressive motor and memory impairments on the rotarod and fear conditioning assays, respectively. At 10 months, their brains displayed somatic BSN and pathological tau accumulation, with the gene expression changes indicating alterations of microglia, classical complement cascade, and synapse pruning pathways, as well as dysregulation of protein quality control pathways. Neuropathological data suggests that the BSN P3866A mutation instigates robust microglia activation, increased microglia‐mediated synapse engulfment, and a co‐deposition of proteasomal subunits with BSN. Furthermore, snRNA‐seq data suggest changes in neuronal populations and cell signaling.

**Conclusion:**

Our BSNKI mouse model revealed the contributions of BSN P3866A mutation to tau pathogenesis and neurodegeneration. BSNKI animals displayed progressive motor and memory impairments, deposits of endogenous tau and BSN, as well as changes in gene expression and neuronal populations. Notably, BSN likely plays a dual role, promoting tau aggregation and sequestering protein degradation molecules, leading to tau and protein accumulation at the soma and synapse, which in turn could trigger neuroinflammation and other cellular changes. These findings propose BSN as a promising therapeutic target for tauopathies, underscoring the need for further exploration to elucidate underlying mechanisms and therapeutic implications.

